# Repercussions of intraalveolar placement of combination of 0.2% chlorhexidine & 10 Mg metronidazole gel on the occurrence of dry sockets- A randomized control trial

**DOI:** 10.4317/jced.53262

**Published:** 2017-02-01

**Authors:** Jasleen Kaur, Rushik Raval, Anupam Bansal, Vinod Kumawat

**Affiliations:** 1 Postgraduate student, Dept of Oral and maxillofacial surgery, Maharaja Ganga Singh Dental College and Research Centre, 11, LNP, Hanumangarhroad, Near RICCO, Sri Ganganagar, Rajasthan, India; 2 Assistant Professor, department of oral and maxillofacial surgery, Darshan Dental college, Udaipur

## Abstract

**Background:**

To evaluate the effects of intraalveolar placement of gel containing 0.2% chlorhexidine and 10gm of metronidazole on the incidence of alveolar osteitis.

**Material and Methods:**

A total of 300 impacted third molars were extracted in 150 patients enrolled in this trial. In each subject a socket was randomly selected and packed to the crest of alveolar ridge with the gel. The contralateral socket was packed with placebo dressing. The occurrence of dry socket was assessed during 3rd and 5th postoperative days .The data was analysed using a meta analytical program.

**Study Design:**

Double blind, prospective, placebo controlled trial.

**Results:**

The combination of metronidazole + chlorhexidine gel significantly reduced dry socket incidence from 22.6% to 6.6% (*P* ≤ 0.001) [McNemar and chi-square tests].

**Conclusions:**

The decrease in incidence of adverse reactions and complications related to local application of metronidazole and chlorhexidine gel explains its clinical use, specifically in mandibular molar extractions where the chances of dry sockets are high.

** Key words:**Chlorhexidine, dry socket, intra-alveolar, metronidazole, placebo.

## Introduction

Removal of impacted third molar is the most consistently performed procedure in oral and maxillofacial surgery. A diverse array of complications have been found associated with lower third molar removal for instance pain, swelling, trismus, inflammation or nerve damage (1). The complication of utmost concern in the field is “Dry socket” which has its onset between 2-4 days after surgery (2). The term was first coined by Crawford (3) Later Brin labelled it as fibrinolytic alveolitis (4-6). It is an inflammatory situation of bone with characteristic traits like throbbing pain, a socket either partially or completely void of blood clot, exposure of bone and erythema of the surrounding gingiva (7). The name dry socket is used since the socket has a dry appearance as the blood clot gets faded and washed away. The frequency of occurrence is ten times more in mandible than in maxilla. It varies from 1 to 4% reaching upto 46% for mandibular third molars (8,9).

The exact etiology of alveolar osteitis is not well understood. Brin suggested that increased fibrinolysis lead to disintegration of clot which is responsible for alveolar osteitis. The alveolus empties, the osseous surroundings are denuded and covered by yellow, grey necrotic tissue layer and the surrounding mucosa becomes erythmatous. According to Fazakerlev and Field (9). It is characterized by stern, debilitating, unalterable and constant pain that continues throughout night. Poor oral hygiene and ensuing alveolar contamination is also an important factor for the onset of dry socket. Microorganisms have been found associated with dry socket such as streptococcus alpha and beta haemolyticus and trepenoma denticola, 70% of them are aerobic and 30% anaerobic (10).

The treatment of paramount importance for dry socket is prevention. Methods advocated for this purpose are antibiotics (Placement in wound, topical antiseptic rinses, antifibrinolytic agents, warm saline gargles,occlusive dressings etc, (11). Since microorganisms are involved in the etiology of alveolar osteitis, the effective treatment approach is application of antibiotics and antiseptics. Preoperative chlorhexidiene mouthwashes have been moderately effective, systemic antibiotics have shown mixed results but combination of both have shown promising results (1,12).

To prevent the problems caused by dry socket persuaded us to opt for the present study. Its aim was to develop a straightforward, uncomplicated, easily administrable treatment which could safely halt the development of dry socket and could be used by all the dental practitioners. It was important that it should be quick and simple to administer, did not require extensive preoperative preparation or the use of systemic antibiotics. Metronidazole and chlorhexidine were selected because of their good safety profile, low risk allergy and effective against pathogens that cause oral infections.

Metronidazole is a nitroimidazole antiinfective agent which has specific activity against a number of anaerobic organisms. It is bactericidal in nature. The exact mechanism of action has not been well eluciadated. It is seen that it is reduced by low redox potential electron transfer protein eg. ferrodoxin to an unidentified polar products which lack the nitro groups. The reduction product appears to be cytotoxic and has antimicrobial effects by disruption of DNA and inhibition of nucleic acid synthesis. Many authors have found metronidazole alone to be ineffective (13). On the contrary chlorhexidine is a biguanide antiseptic used as a mouthwash or bioadhesive gel. It is active against a wide variety of aerobic/ anaerobic oral pathogens. It is shown to be effective in prevention of dry socket by some authors whereas some find it to be ineffective (11,14-17).

Intralveolar placement is advantageous as it allows for more bioavailability and thus more prolonged release of active substance and more direct action on alveolus hexidiene (14-16). At present, as there hasn’t been much information about the use and effects of these two drugs in combination especially as local application, this present study aims to assess the effects of intraalveolar application of chlorhexidine and metronidazole gel on the incidence of alveolar osteitis which is first time reported of its kind of studies.

## Material and Methods

This study was performed on 150 patients requiring bilateral extraction of mandibular third molars. The inclusion criteria were patients between 20 to 45 years of age. The exclusion criteria includes: unwilling to participate, reluctant to avoid consuming antibiotics, failure to attend follow up sessions, presence of physiological condition or receiving sedatives, any need for antibiotic prophylaxis, pregnancy, allergy to medication. The ethics were approved and complications of the study were explained to the patient prior to surgery. Informed consent was obtained.

-Operative technique

The procedure was undertaken under local anaesthesia (2% lignocaine + adrenaline 1: 80,000). Inferior alveolar, lingual and buccal nerve blocks were given and after desired incision, full thickness mucoperiosteal flap was reflected. Osteotomy and odontectomy were carried out whenever necessary. After removal of tooth the socket was debrided and irrigated with normal saline, bone edges were smoothened. After extraction, another operator randomly chose the socket in which experimental and placebo dressing was to be placed. The wound was then sutured with 3-0 black braided silk sutures and the patient was prescribed analgesic (aceclophenac + serratiopeptidase ) twice daily for 3 days.

-Diagnosis and collection of data

The occurrence of dry socket was then evaluated on 3rd & 5th postoperative days. The presence of any of the 2 criteria indicated dry socket-throbbing pain not relieved by analgesics, presence of dark partially resorbed blood clot on irrigation, pain which relieved significantly on application of eugenol dressing.

-Analysis of Data

The significance of differences among the data was calculated with the help of SPSS (version 17, SPSS Inc, Chicago, IL, USA) was done to calculate the incidence of alveolar osteitis. McNemar and chi-square tests were applied and *p*-value was obtained.

## Results

**Figure 1:**
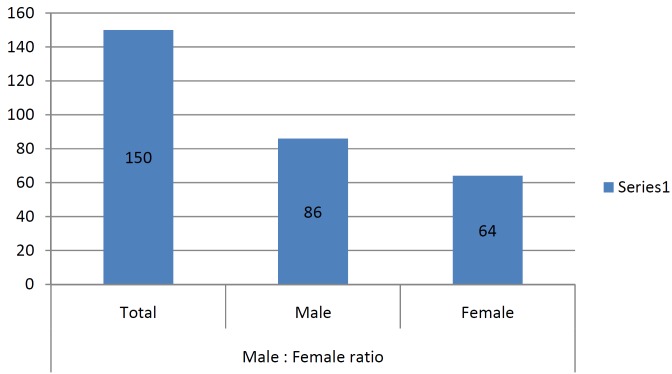
Male: Female ratio.

**Figure 2:**
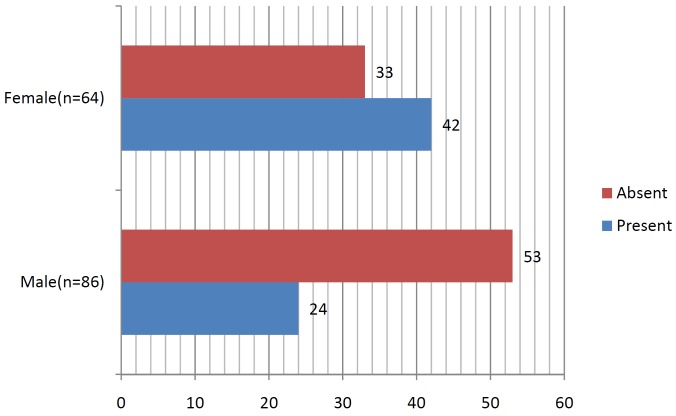
Occurrence of dry socket amongst male and female on control side.

A total of 150 patients were studied and the mean age being 30.5+/- 2.5, out of which 42.6% were females and rest 57.3% males ( Table 1, Fig. 1). The proportions of dry socket in the control and experimental sides were 22.6% and 6.6% respectively. On control side 15 out of 86 males i.e. 17.4% patients developed dry socket and 19 out of 64 i.e. 29.6% female patients developed dry socket ( Table 2, Fig. 2). A total of 10 patients (4 males and 6 females) developed bilateral alveolar osteitis.

**Table 1:**
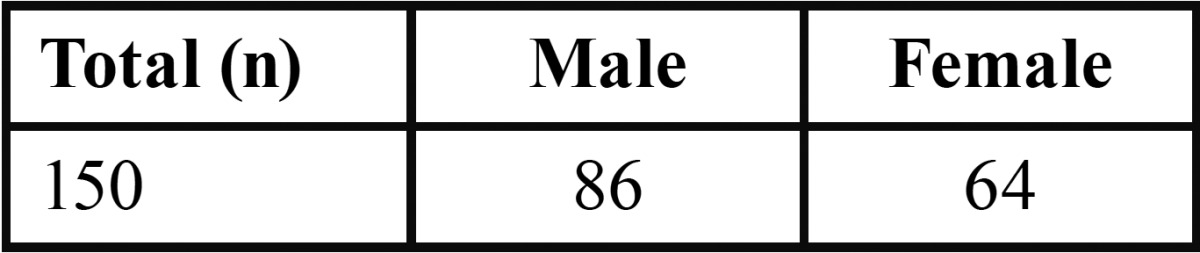
Male: Female ratio.

**Table 2:**
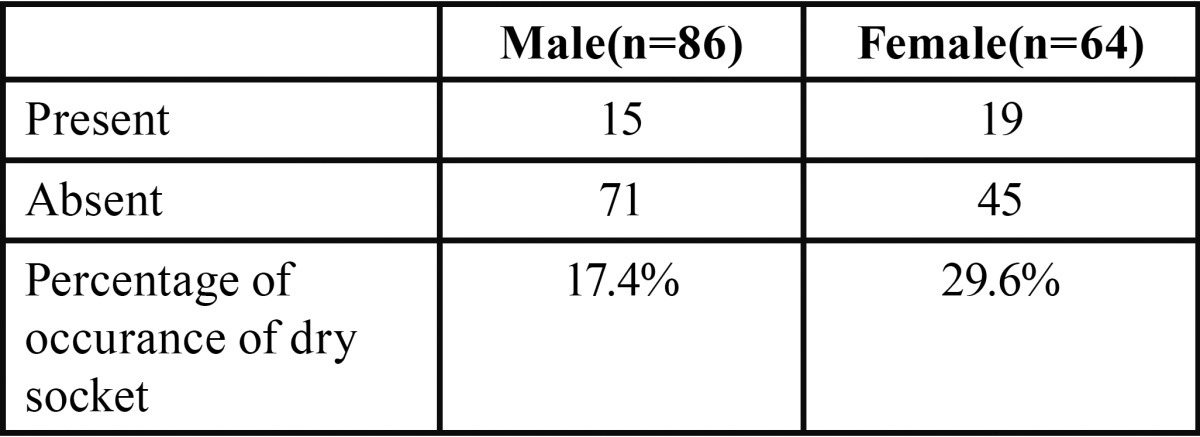
Incidence of dry socket on control side.

**Figure 3:**
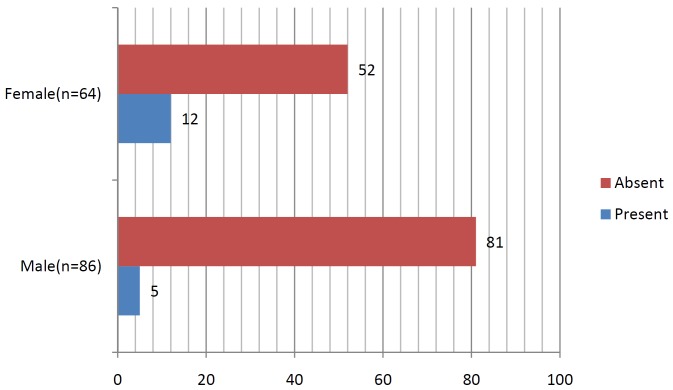
Occurrence of dry socket amongst male and female on the case side.

On experimental side, where metronidazole and chlorhexidine dressing was placed only 2 out of 86 males i.e. 1.72% patients developed dry socket and 8 out of 64 females i.e. 9.37% patients developed dry socket ( Table 3, Fig. 3). When alveolar osteitis was absent on control side, it was also absent on experimental side. The level of significance for all the tests was set at *P* = 0.05 ( Table 4).

**Table 3:**
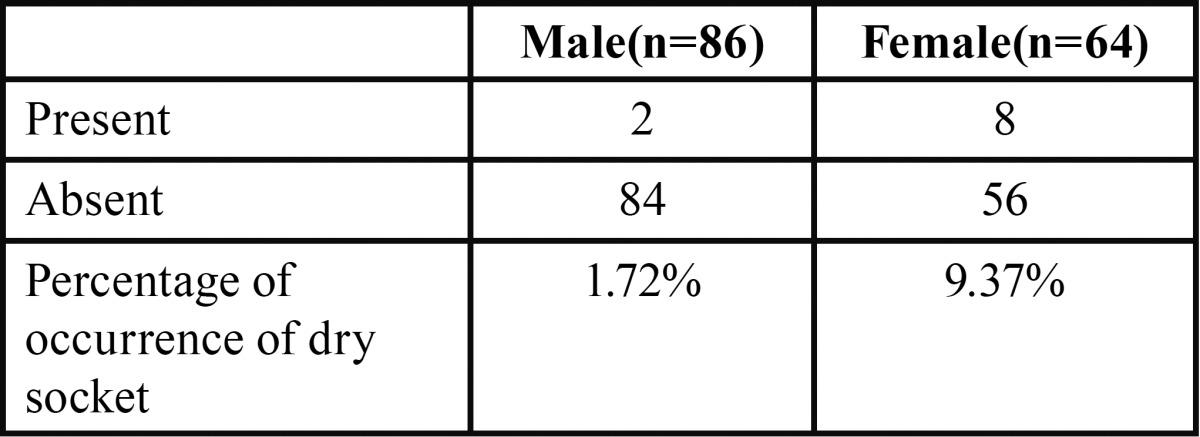
Incidence of dry socket on case side.

**Table 4:**
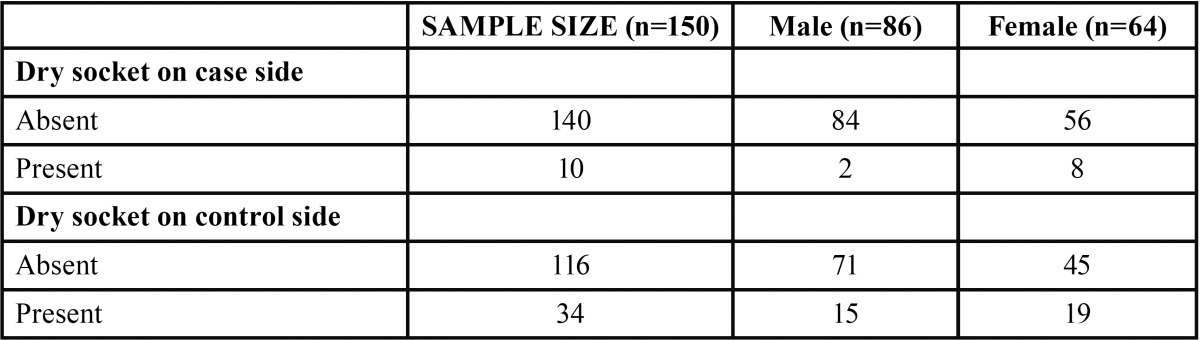
Incidence of dry socket amongst case and control group.

## Discussion

Alveolar osteitis is not considered a disease of bacterial infection but a healing disturbance due to loss of blood clot in extraction socket. Bacterial invasion is most likely one of the many factors contributing to blood clot disintegration and development of alveolar osteitis. Surgical trauma, age, gender are other known risk factors for the development of alveolar osteitis and other postoperative complications. Because oral surgery is always carried out in a clean contaminated environment where a large number of bacteria exist and as said postoperative complications are usually associated with bacterial contamination, it seems reasonable to use antibiotics to reduce the frequency of complications. The role of bacteria in alveolar osteitis has long been postulated. There has been increased frequency of dry socket in patients with poor oral hygiene, pre-existing local infection such as pericoronitis and advanced periodontal disease. Nitzan *et al.* showed the possible significance of anaerobic micro organisms in relation to etiology of dry socket (10).

The results of this meta analysis of randomised controlled trials indicated that application of topical metronidazole and chlorhexidiene gel is statistically significant in reducing the frequency of alveolar osteitis after transalveolar extraction of impacted third molars. The bactericidal effects of metronidazole on these microorganisms and the antiseptic effects of chlorhexidine on bacteria inducing fibrinolysisis thought to be responsible for positive results of the study. In this study the male:female ratio did not differ significantly. Smokers and people using alcohol were not included in this study. There was a marked difference in the incidence of dry socket amongst male and female both in the case and control side. The varying results may be due to estrogen level fluctuations. It enhances the fibrinolytic activity. Therefore additional estrogen in the form of oral contraceptives may increase the chances of dry socket in women. Although some studies have failed to find such association (18). Most of the male patients who reported with the history dry socket may have smoked even after proper postoperative instructions which could be the probable cause of dry socket.

Many studies have been executed on the topical use of antibiotics to treat dry socket. Haraji *et al.* in their study on effects of intra-alveolar placement of 0.2% chlorhexidine bioadhesive gel on dry socket incidence and postsurgical pain concluded that topical chlorhexidine gel significantly reduce dry socket incidence and besides that it also reduce postsurgical pain in patients with and without dry socket (11). Torres-Lagares D *et al.* in their pilot study, concluded that the bio-adhesive 0.2% chlorhexidine gel when applied only once after the extraction of impacted third molars seems to be an appropriate option for the reduction of alveolitis. It improves the buccal aperture and post extraction edema in the post-operative period (14). Other studies of intraalveolar placement of chlorhexidine gel also gave promising results on incidence of dry socket following 3rd molar removal (16,19-21).

Mitchell investigated the efficacy of a gel made up of 10% metronidazole for the treatment of dry socket. He observed faster healing when the gel was used. Since the evident input of anaerobic bacteria in the etiology of dry socket, he recommended the use of nitroimidazoles for the treatment and prevention of dry socket (21,22). Poi *et al.* in his clinical trial after application of topical gel composed of 10% metronidazole, 2% lidocaine, and carboximetilcelulose as the base and mint with 5% ascorbosilane C found that it reduced free radicals, confined the cellular membrane, and regenerated cutaneous tissues, in addition to helping the synthesis of collagen and elastin. From these outcomes, they concluded that the paste was effective in the treatment of infection and did not interfere with the normal chronology of the healing process in an experimental dental model of an infected alveolus in the rat. Thus the ideal dressing for filling the alveolus should be bactericidal, antifibrinolytic, and analgesic and should contribute to alveolar healing (23). Inamdar MN *et al.* in their study on prevention of dry socket using Chlorhexidine Gel and Ornidazole Gel concluded that both chlorhexidine gel and ornidazole gel are effective in reducing post-operative complications like dry socket, pain & swelling after impacted 3rd molar removal (24). Efficacy of chlorhexidine, metronidazole and combination gel in the treatment of gingivitis was studied by AR Pradeep *et al.* and the results showed that significant clinical and microbiological improvement was achieved with local application of gel and thus it may have a role in the management of gingivitis. The use of a bioadhesive gel with the combination of metronidazole and cholrhexidine gel as an intraalveolar medicament has not been reported much in the literature. In the present study a single application of this combination gel was used as an intraalveolar medicament following 3rd molar removal. Hopeful results have been achieved in the incidence of dry socket postoperatively which are concomitant to the findings of the other studies (19-25).

## Conclusions

There seems a clear advantage of intraalveolar medication after extraction. The lack of adverse reactions and complications related to metronidazole and chlorhexidine gel explains its clinical use specifically in mandibular molar extractions where the chances of dry sockets are more and adds some advantages compared to the rinses in terms of increased drug bioavailability and reduction of staining and taste disturbance. This method is cheap, easily available and not time consuming and can be performed simply to provide the patient with significant pain relief caused by dry socket.
